# Erdheim-Chester disease with bilateral orbital masses and multi-systemic symptoms: two case reports

**DOI:** 10.1186/s12957-023-03123-5

**Published:** 2023-07-31

**Authors:** JunYi Qiao, Ruixin Ma, Xiaolin Peng, Weimin He

**Affiliations:** Department of Ophthalmology, West China Hospital of Sichuan University, Sichuan Chengdu, 610041 China

**Keywords:** Non-Langerhans cell histiocytosis, Erdheim, Chester disease, ECD, Inflammatory myeloid neoplasm, *BRAF *^*V600E*^, Primary thrombocytosis, Case report

## Abstract

**Background:**

Erdheim–Chester disease (ECD) is a rare histiocytic disorder characterized by multisystem xanthogranulomatous infiltration by lipid-laden histiocytes. We report two cases of ECD involving the orbit and describe their clinicopathologic factors, treatments, and prognosis. One was a rare case of ECD complicated with primary thrombocytosis.

**Case presentation:**

This study describes two patients with bilateral orbital ECD. Both presented with proptosis and visual loss; imaging findings showed bilateral intraorbital masses. Both had different degrees of systemic symptoms (pleural effusion, pericardial effusion, ascites, and heart failure) before the ocular symptoms and did not find the cause before ophthalmic tumor resection and pathological biopsy. The diagnosis of ECD was confirmed after pathological biopsy and detection of *BRAF*^*V600E*^ mutation. Patient 2 also with primary thrombocytosis and had a *CALR* mutation as well as the *BRAF*^*V600E*^ mutation. Both patients were recommended to receive targeted therapy. Patient 1 refused targeted therapy for financial reasons and was discharged after local radiotherapy only. The patient had no light perception in either eye and no improvement in systemic symptoms. Patient 2 began targeted treatment after diagnosis and reached the discharge criteria 2 weeks later. He is in good condition at present, but unfortunately, his eyesight has not improved because of the irreversible damage to his visual function.

**Conclusion:**

ECD is easily misdiagnosed and missed because of its rarity and diverse clinical manifestations. Orbital involvement is common in ECD, and surgery is the most frequently employed approach. Despite the surgical resection is not curative, its significance lies in biopsy to establish diagnosis and/or surgical debulking to relieve mass effect, minimizing further impairment of visual function. Targeted therapy is the most effective treatment for patients with a positive *BRAF* mutation gene. Evaluation of a concomitant myeloid neoplasm is also critical before initiating targeted therapies for refractory ECD.

## Background

Erdheim–Chester disease (ECD) is a rare histiocytic disorder characterized by multisystem xanthogranulomatous infiltration by lipid-laden histiocytes. The World Health Organization classified ECD as “a provisional entity under histiocytic and dendritic cell neoplasms with B-rapidly accelerated fibrosarcoma (*BRAF*) gene mutations” in 2016 [1]. More than 1500 cases of the disease have been reported in the medical literature [2], but clinicians still do not understand the disease well. Not only because of its highly varied clinical manifestations and course of the disease, ranging from isolated focal lesions to life-threatening multiple-organ failure but also because of the rarity of the disease, the evaluation of therapeutic effectiveness has been based on small-scale studies or case reports.

We report two cases of ECD involving the orbit. The two cases highlight the different clinical manifestations, classic radiological and histopathological findings, and differences in response to treatment. One patient had a rare case of ECD complicated with primary thrombocytosis. Roughly 10.1% of ECD is associated with myeloproliferative diseases, and these two diseases may share some common molecular mechanisms [3].

## Case presentation

### Case 1

**Fig. 1:**
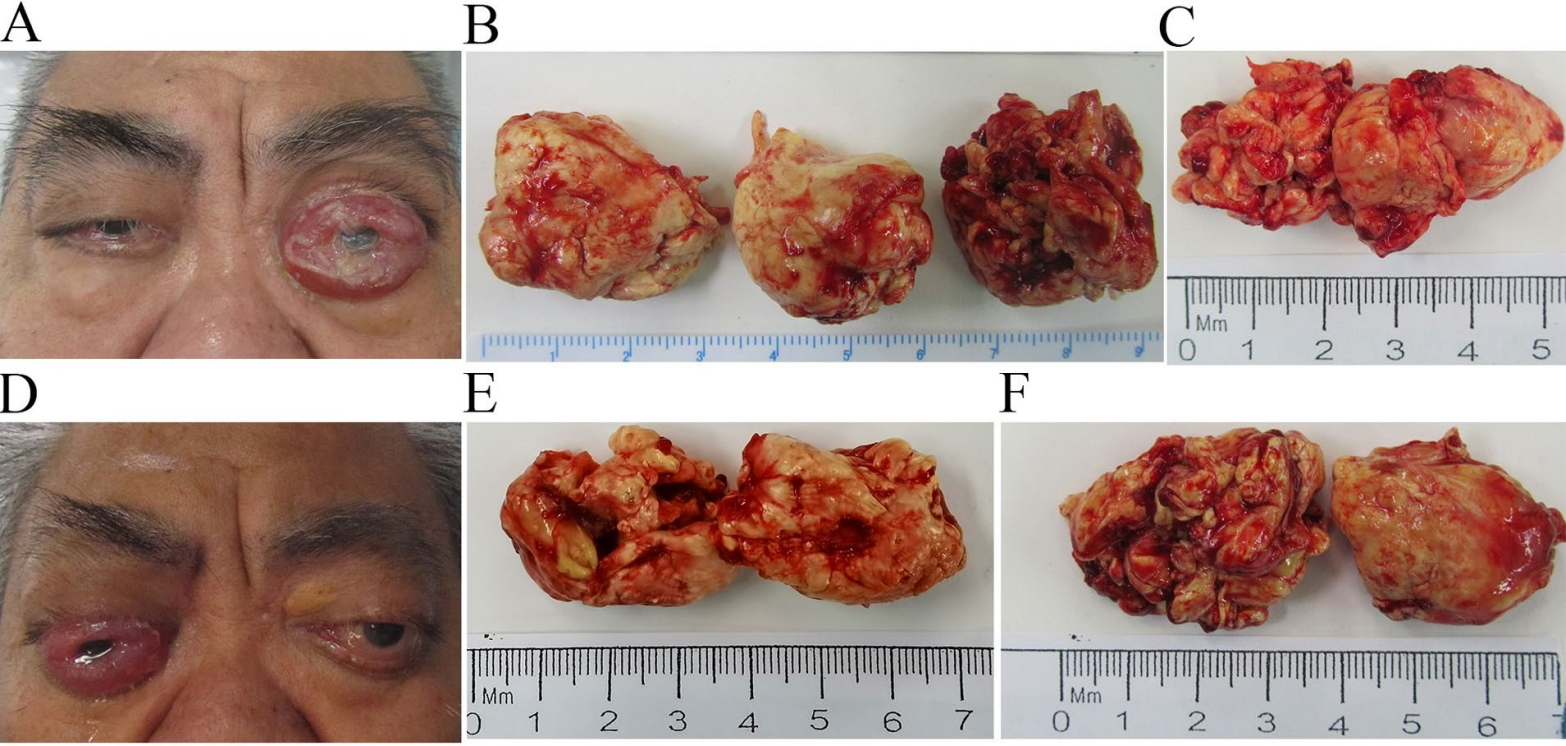
**A** The patient’s picture before the first operation showed prolapse of the conjunctiva of the left eyeball. **B** The left orbital mass was removed during the first operation: the hard mass filled the orbit and surrounds the optic nerve. **C** Mass in the right orbit during the first operation. **D** Conjunctival edema of the right eyeball occurred again 1 year after the first operation. **E** In the second operation, the mass was removed from the left orbit. **F** In the second operation, the mass was removed from the right orbit

**Fig. 2:**
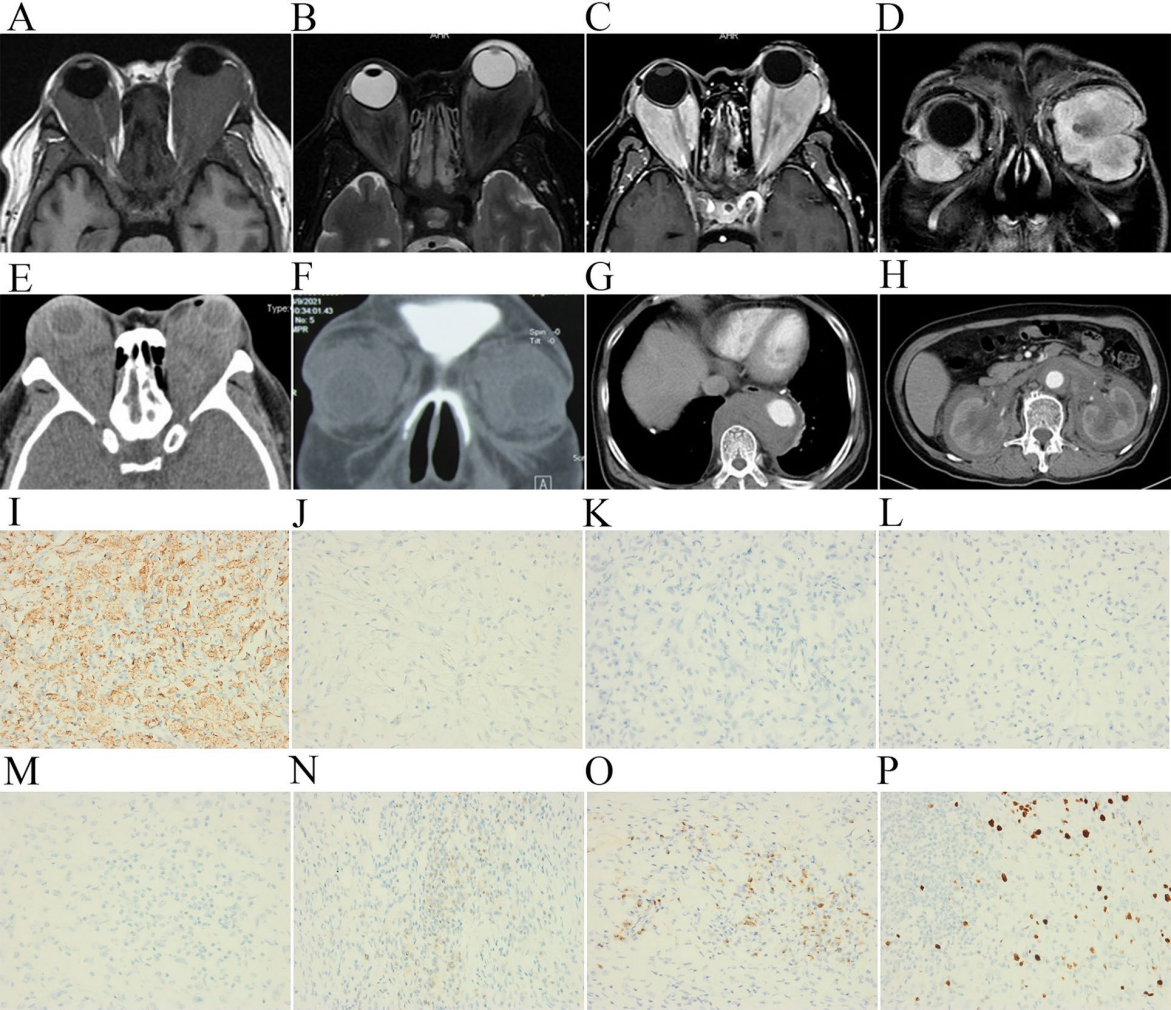
**A**–**D** Before the first operation, enhanced MRI of the orbit. Bilateral retrobulbar lesions filled with equal T1 and T2 signals and uneven enhancement on the enhanced scan. The mass surrounding the bilateral optic nerve and bilateral eyeball compression protrusion is more obvious on the left than on the right. **E**, **F** A year later, CT of the orbit revealed soft tissue density filling behind the bilateral intraorbital bulbs. **G**, **H** CT demonstrates pericardial thickening and effusion and bilateral hydronephrosis with peri-nephric fat stranding. **I** Positive cytoplasmic CD68 immunostaining of tumor cells (CD68, original magnification × 400). **J** Negative cytoplasmic S-100 immunostaining of tumor cells (S-100, original magnification × 400). **K** Negative cytoplasmic Langerin immunostaining of tumor cells (Langerin, original magnification × 400). **L** Negative cytoplasmic CD1a immunostaining of tumor cells (CD1a, original magnification × 400) **M** Negative cytoplasmic ALK immunostaining of tumor cells (ALK, original magnification × 400). **N** Positive cytoplasmic CD138 immunostaining of tumor cells (CD138, original magnification × 400). **O** Positive cytoplasmic IgG4 immunostaining of tumor cells (IgG4, original magnification × 400,  > 100 cells/HPF). **P** Ki67 immunostaining demonstrating that approximately 10–20% of tumor cells showed strong nuclear immunoreactivity (Ki67, original magnification × 400)

A 57-year-old male was admitted to our department with chronic headaches and bilateral visual loss for over 2 years and recurrent pain and swelling around his left eye for 10 months. Before admission, he was diagnosed with “left orbital cavernous haemangioma” in another hospital, which was treated with a gamma knife 2 years ago, but the symptoms had not improved. He had a 1-year history of heart failure and pulmonary infection. Our examination showed proptosis, restriction of eye movements, eyelid insufficiency, bulbar conjunctival congestion and edema, and a corneal ulcer in his left eye (Fig. 1A). There was conjunctival congestion and edema, restriction of eye movements, and high intraocular pressure (28 mmHg) of the right eye, with no other positive findings. His vision was 1.0 in the right eye, but his vision in the left was hand movement (HM). Orbital MRI showed bilateral intraorbital masses, and the left orbit was more evident (Fig. 2A–D); a whole-body CT demonstrated ascites, pleural effusion, pericardial thickening and effusion, and bilateral hydronephrosis with peri-nephric fat stranding (Fig. 2G–H). Echocardiography findings revealed right ventricular enlargement, left ventricular hypertrophy, pulmonary hypertension, and a widened aorta and pulmonary artery. A blood count showed normocytic anemia (hemoglobin concentration 98 g/L), high platelet counts (PLT 536 × 10^9^/L), and normal leukocytes. Biochemical studies showed hypokalaemia (K^+^ 2.81 mmol/L) and hypoproteinaemia (albumin 33.7 g/L). The patient underwent surgery on both eyes, and the tumor was removed as completely as possible (Fig. 1B, C). Orbital biopsy showed fibrous tissue hyperplasia with chronic inflammatory cell infiltration and many histiocyte reactions, with a pathological diagnosis of inflammatory pseudotumor. The bulbar conjunctival edema improved, the corneal ulcer subsided, and the eyesight improved in his left eye 2 weeks after the operation.

Unfortunately, the patient was admitted to the hospital again a year later because of recurrent pain and swelling around his right eye for half a month. Physical examination showed restriction of eye movements and corneal macula in both eyes as well as high conjunctival hyperemia and edema of the right eye (Fig. 1D). His vision was 0.02 in the right eye and light perception (LP) in the left eye. Orbital CT showed bilateral intraconal masses (Fig. 2E, F). Left ventricular hypertrophy and a widened aorta and pulmonary artery were still observed by echocardiogram. The patient still had hypoproteinaemia and hypokalaemia. The bilateral orbital masses were surgically resected again (Fig. 1E, F), and the pathology was diagnosed as non-Langerhans cell histiocytosis with *BRAF* gene mutation (Fig. 2I–P). An abundant IgG4-positive plasma cell population was detected (> 100 cells/HPF) in the tissue locally, but the IgG4 level in the serum was average. Despite the differential diagnosis of IgG4-related disease, a diagnosis of ECD was made because of the positive *BRAF*^*V600E*^ mutation. We recommended the *BRAF* inhibitor vemurafenib for treatment. The patient rejected the plan because of financial problems and discharged himself from the hospital after local radiotherapy. At his most recent follow-up in Feb. 2023, he had no recurrence of orbital tumors but his systemic symptoms had not improved.

### Case 2

A 65-year-old male presented to our hospital for management of severe bilateral proptosis, vision loss, eye pain, and edema of both lower limbs. Five months ago, he was diagnosed with “hypoalbuminaemia” in another hospital because of edema of both lower extremities, and it improved after treatment with an albumin supplement. Then, he gradually had proptosis, vision reduction, and eye pain over the next month. At the same time, he began feeling dyspnea, decreased exercise tolerance, and shortness of breath after a short exercise. After several check-ups, he was diagnosed with hypoproteinemia and polyserositis; after treatment with an albumin supplement and prednisone (50 mg PO qd), the symptoms improved. However, his symptoms recurred once he began tapering the prednisone. One week before admission, the patient had a sharp decline in binocular visual acuity and eye pain. He had a 3-year history of primary thrombocytosis (next-generation sequencing revealed *CALR* mutations).

**Fig. 3:**
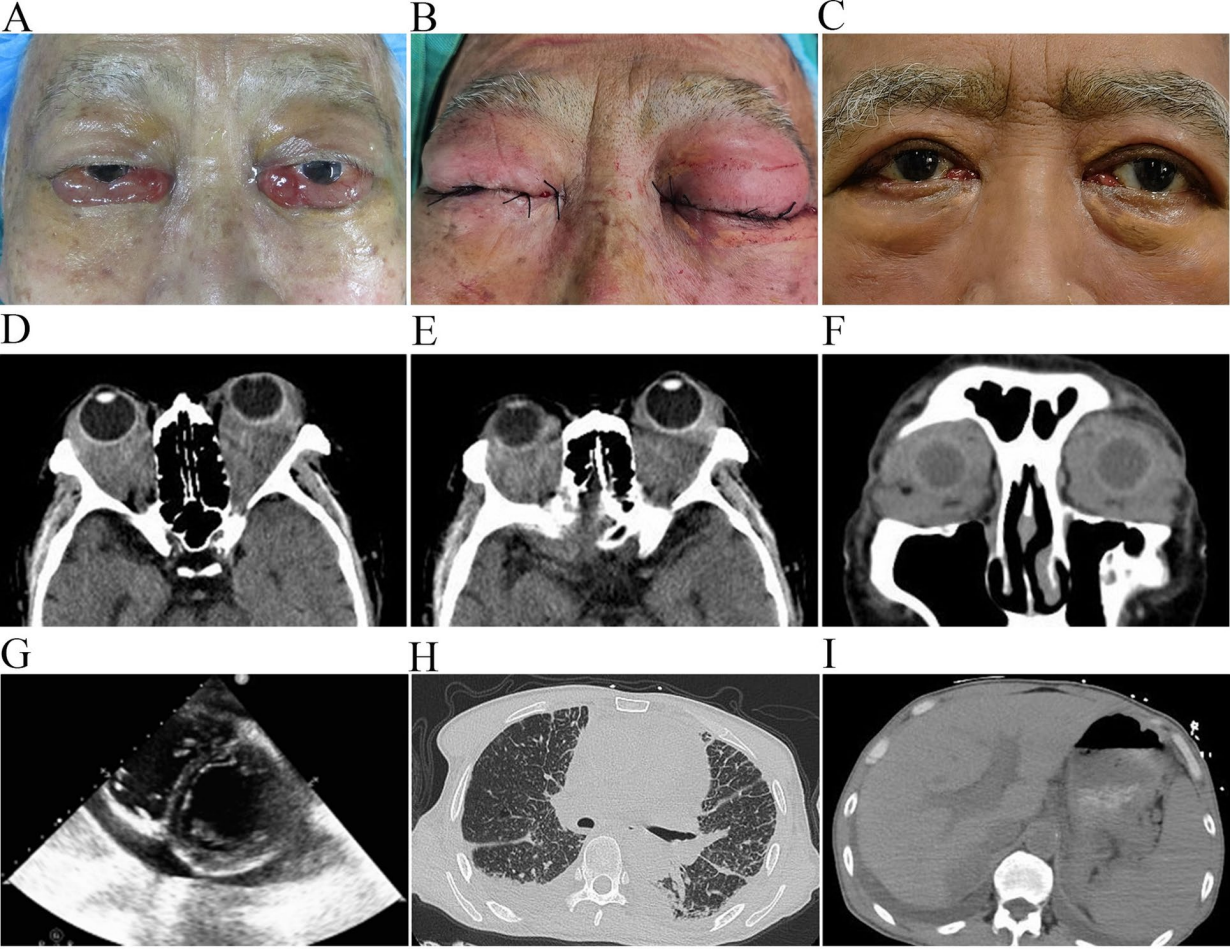
**A** Proptosis, bulbar conjunctival congestion, and edema of both eyes. Xanthelasma was noted around both eyes. **B** Eyelid swelling postoperatively. **C** After treatment with vemurafenib, the patient returned to normal overall, but vision recovery was impossible. **D**–**F** Orbital CT. A soft tissue density shadow can be seen in the bilateral orbit, which partially surrounds the eyeball and optic nerve. **G** Pericardial effusion and left ventricular hypertrophy were observed by echocardiogram. **H** CT showed pleural effusion. **I** CT showed ascites and swelling of the kidney

The examination showed proptosis, restriction of eye movements, bulbar conjunctival congestion and edema, and high intraocular pressure in both eyes (Fig. 3A), and xanthelasma was noted around both eyes. His visual acuity showed no light perception (NLP). Orbital CT showed bilateral intraconal masses (Fig. 3D–F). A blood count showed high platelet counts (PLT 671 × 10^9^/L). Biochemical studies showed hypokalaemia (K^+^ 3.32 mmol/L) and hypoproteinaemia (albumin 26.6 g/L). Whole-body CT and 18F-FDG PET/CT showed pleural effusion, pericardial effusion, and ascites (Fig. 3H, I). An echocardiogram revealed left ventricular hypertrophy, a widened aorta and pulmonary artery and severe pericardial effusion (Fig. 3G). Pericardiocentesis was performed. Microbiological tests on the harvested pericardial effusion were negative.

**Fig. 4:**
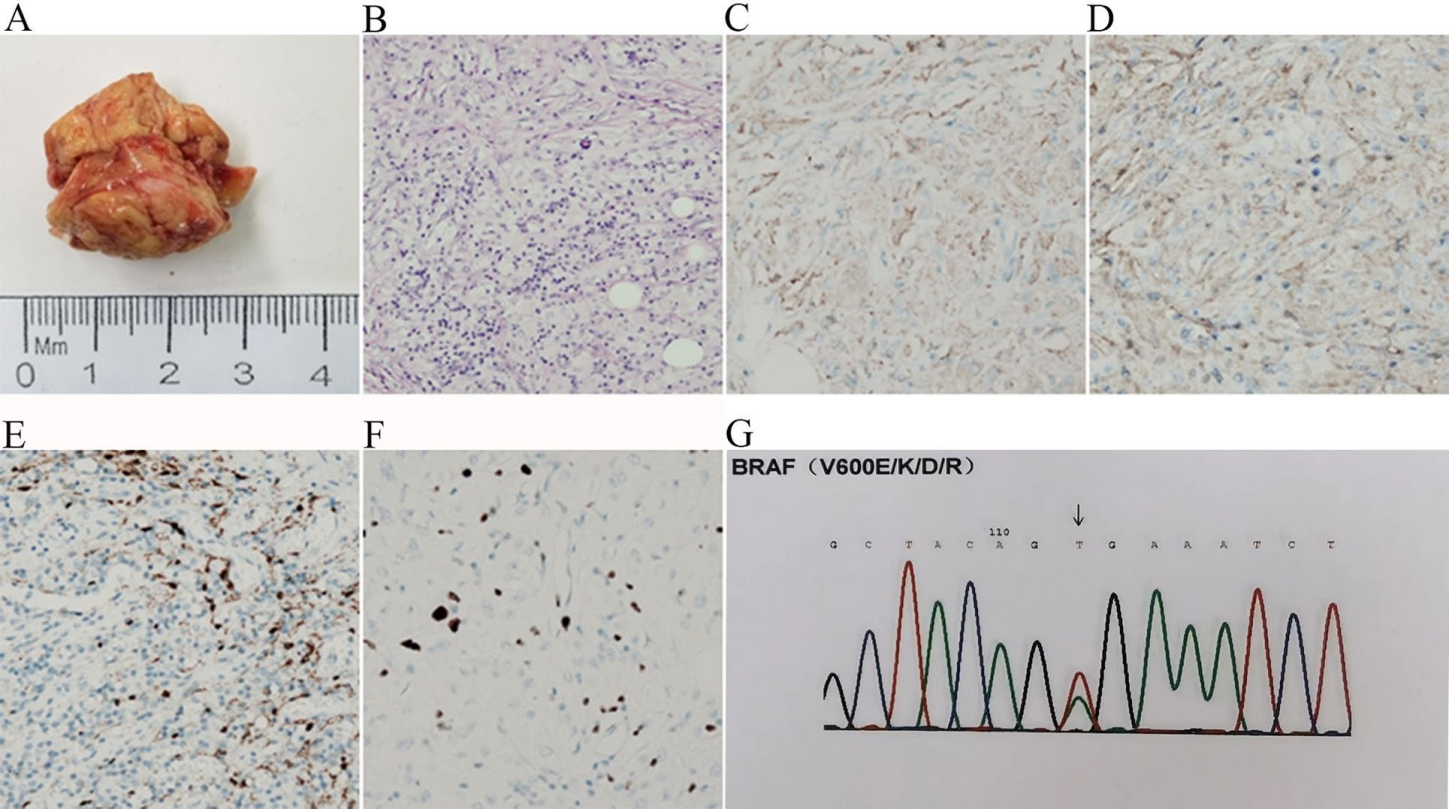
**A** Gross resection specimen of the left orbit. The mass is solid and medium in texture; its section is grayish white and greyish yellow. **B** Histiocytic proliferative lesion, nodular/patchy nest conformation. Collagen fibers and fat vacuoles are in the background (hematoxylin–eosin, original magnification × 200). **C** Positive cytoplasmic CD68 immunostaining of tumor cells (CD68, original magnification × 400). **D** Positive cytoplasmic CD163 immunostaining of tumor cells (CD163, original magnification × 400). **E** Negative cytoplasmic S-100 immunostaining of tumor cells (S-100, original magnification × 400). **F** Ki67 immunostaining: 10–20% of tumor cells showed strong nuclear immunoreactivity (Ki67, original magnification × 400). **G** *BRAF* mutation detection: mutation in exon 15 was detected (V600E)

To confirm the diagnosis of the disease, an orbital biopsy was arranged (Fig. 4A), which revealed the adipose tissue infiltrated by foamy histiocytes that were CD68 positive, including multinucleated giant cells/Touton cells and scattered lymphocytes (Fig. 4B–F). This constellation of clinical manifestations and histopathological findings led to a diagnosis of multisystem ECD. The patient received further treatment in the intensive care unit (ICU) after the operation due to the patient’s poor systemic symptoms. He started treatment with the *BRAF* inhibitor vemurafenib (240 mg PO bid) because of a positive *BRAF*^*V600E*^ mutation (Fig. 4G), with immediate improvement in his systemic symptoms. What is more delightful was that he reached the discharge standard after 2 weeks of treatment with a *BRAF* inhibitor. However, the patient’s eyesight deteriorated to no light perception (NLP) before treatment, and his visual acuity did not improve. Vemurafenib (240 mg PO bid) was continued for 1 year. Follow-ups showed no recurrence of ocular or systemic symptoms. However, his eyesight remained NLP in both eyes because of the irreversible damage to his visual function, and he needed to take hydroxyurea to control the primary thrombocytosis. It is noteworthy that the patient reported bone and joint pain following medication administration, which was alleviated with non-steroidal anti-inflammatory drugs.

## Discussion and conclusion

The clinical manifestations of ECD range from isolated focal lesions to life-threatening multiple-organ failure and the radiological findings of ECD are specific. And a summary of the characteristic features and ophthalmologic findings of ECD is presented in Tables 1 and 2.

**Table 1:** Characteristic features of Erdheim-Chester disease

Organ involvement	Frequency	Characteristic feature
Bones	95%	Osteosclerosis at the metadiaphysis of bones around the knee, axial skeleton, and small bones of the feet [4]
Endocrine	50–70%	Diabetes insipidus, anterior pituitary hormonal deficiencies, and hyperprolactinemia [5]
Arterial	50–80%	Periaortic infiltration “coated aorta”; infiltration of the supra-aortic trunk branches, visceral arteries, renal artery stenosis, and coronary arteries [6]
Respiratory	50%	Mediastinal infiltration; pleural, septal, and maxillary sinus thickening [6]
Cardiac	40–70%	Pericardial infiltration and effusion, right atrial pseudotumor, and atrioventricular sulcus [7]
Retroperitoneum	40–50%	Perinephric infiltration “hairy kidneys”; hydronephrosis and adrenal infiltration [4]
Nervous system	40%	Cognitive impairment, ataxia, and peripheral neuropath; brainstem/cerebellum masses; cerebral white matter enhancement; and dural and pituitary stalk thickening [6]
Orbit	30%	Orbital masses [3]
Dermatologic	25%	Xanthelasma-like lesions around the eyes, face, neck, and inguinal folds [8]
Testes	3–5%	May occur as a hybrid/overlap disease with RDD [5]

*RDD* Rosai-Dorfman disease

**Table 2:** Ophthalmologic findings of Erdheim-Chester disease

Eye involvement	Characteristic features	Radiological findings
**Palpebrae**	Xanthelasmas	Hypointense on MRI [9]
**Orbit**	Progressive bilateral proptosis [3]	Hypodensity on CT and isointensity to hypointensity on MRI with heterogeneous enhancement [10]
Optic nerve	Decreased visual acuity [3]	Enhancement or compression of the optic nerve on MRI [11]
Extraocular muscle	Ophthalmoplegia [3]	Enhancement or enlargement of the muscle on MRI [11]
**Intraocular**		
Anterior chamber	Uveitis (cells)	——
Vitreous	Vitritis	——
Retina	Recurrent Serous Retinal Detachment [12]	Drusen-like deposits of histiocytic material with progressive staining on fluorescein angiography [12]
Optic disc	Oedema [11]	——
RPE	Chronic pigmentary and atrophic changes along with pigment clumping [12]	——
Choroid	Quiescent choroidal neovascularization [13]	Disappearance of intermediate and large choroidal vessels and intact choriocapillaris in the lesion on EDI-OCT [14]

*RPE *Retinal pigment epithelium, *EDI-OCT *Enhanced depth imaging-optical coherence tomography

The diagnosis of ECD relies on typical radiological findings combined with a site-specific histopathological examination, particularly for detecting *BRAF* mutation or other MAPK pathway alterations [15]. Ocular involvement occurs in approximately 30% of ECD cases [4], and the ocular region offers a superficial and relatively accessible site for obtaining pathological specimens, making it optimal for diagnosis [12]. Thus, surgical intervention to obtain tissue samples for definitive diagnosis is crucial.

The gross resection specimen of ECD is typically a greyish-yellow or greyish-white, tough mass that adheres closely to the tissue. The histological feature of ECD is mononuclear foam tissue cell infiltration, multinucleated giant cells or Touton cells are often seen, and fibrosis can be seen in most cases [16]. Immunohistochemistry exhibits positive expression of CD68 and negative expression of CD1a and S-100 [12]. *BRAF*^*V600E*^ mutation testing (up to 54% of ECD patients have *BRAF*^*V600E*^ mutations) is crucial for diagnosis and effective treatment [12]. This mutation is the most common mutation in cerebral, cardiac, and orbital ECD [17]. But whether the diagnosis of ECD can be doubted usually depends on the pathologist’s experience. In our case 1, the pathological biopsy showed fibrous tissue hyperplasia with histiocyte reactions, no further immunohistochemistry and mutant gene detection were carried out, and the diagnosis of inflammatory pseudotumor was made. It was not until a year later, after the second biopsy, that a diagnosis of ECD was established. In contrast with the experience of case 1, the orbital biopsy in our second case revealed adipose tissue infiltrated by foamy CD68-positive histiocytes. This combined with clinical and imaging findings led quickly to the diagnosis of ECD.

However, it is difficult to distinguish these pseudotumoral lesions. The differential diagnosis of orbital ECD is centered on IgG4-related orbital inflammation, granulomatosis with polyangiitis (GPA), orbital lymphoma, and idiopathic orbital inflammatory disease [18, 19]. These have many overlapping clinical manifestations (orbital masses, retroperitoneal infiltration, periaortitis, and so on) [20] with ECD. Typical long bone lesions and xanthelasma-like lesions are unique clinical manifestations of ECD, but there are also many atypical cases. IgG4-positive plasma cells are always detected in the pathological tissue of ECD of the orbit [21], making it more difficult to distinguish it from IgG4-related disease (IgG4-RD). In case 1, an affluent IgG4-positive plasma cell population was detected, but *BRAF* showed a mutation in exon 15, confirming the diagnosis of ECD. Therefore, tissue biopsy by an experienced pathologist and identification of *BRAF* and other MAPK pathway mutations in biopsies are key in diagnosing the disease [6].

Treatment options for ECD include corticosteroids, biologic agents (IFN-α), chemotherapy, and BRAF inhibitors (BRAFIs) [15]. Traditional approaches like IFN-α are recommended for patients with mild-to-moderate disease severity [2]. And surgery is crucial for ECD-involved orbit, serving not only for diagnostic biopsy but also for relieving mass effect, minimizing further impairment of visual function. The mass exerts pressure on critical structures, such as blood vessels and nerves, resulting in irreversible visual impairment. And proptosis and exposure keratopathy can also occur due to the presence of an intraorbital mass, impacting visual function and causing discomfort. Due to the diffuse growth pattern observed in ECD, surgical excision presents significant challenges [22] and only when the tumor reaches a considerable size will it begin to develop ocular symptoms such as proptosis and visual decline. Therefore, early screening and timely treatment of ocular involvement when patients present with symptoms involving other organs or systems can maximize the preservation of visual function.

In *BRAF*^*V600E*^ patients, BRAFIs are recommended as the first-line therapy [2]. Vemurafenib, an FDA-approved BRAF inhibitor for ECD treatment [23], shows rapid improvements in radiology, clinical outcomes, and laboratory markers within 4 weeks for refractory ECD patients [24]. Just like in Case 2, the patient experienced significant improvement in symptoms within 2 weeks of medication use. The standard dose of Vemurafenib is 960 mg twice daily, but lower doses of 480 mg have shown good tolerability [12]. Recent studies by Ruan and Saunders et al. also suggest that lower doses (25–50% of the FDA-approved dose) are both effective and well tolerated and are recommended as initial treatment [25, 26]. In case 2, the patient received a starting dose of 240 mg/bid (25% of the FDA-approved dose) for 1 year and no recurrence of symptoms during follow-up. But the patient started experiencing bone and joint pain after taking the medication, which we speculate to be a side effect of Vemurafenib. This is consistent with the known side effects of Vemurafenib, which include arthralgias, pancreatitis, QT prolongation, and cutaneous manifestations such as squamous cell carcinoma and keratoacanthoma [27]. Patient 1 had the *BRAF*^*V600E*^ mutation, but he rejected targeted therapy for financial reasons. The patient underwent surgery and local radiotherapy; he had no recurrence of orbital tumors within 1 year after treatment, but his polyserositis did not improve. Although complete remission could not be achieved, it seemed he made a good choice. ECD is not a radiosensitive disease, but radiotherapy is a good method for situations where immediate palliation of symptoms is needed (large tumors causing CNS, ocular, or internal organ compromise) [2, 28].

The existence of both ECD and primary thrombocytosis in our case 2 is interesting. Histiocytic and dendritic cell neoplasms can be clonally related to leukemias [29, 30]. Approximately 10.1% of patients with ECD have an overlapping myeloid neoplasm [31] and hallmark driver mutations of myeloid neoplasms (such as *JAK2V617F* and *CALR* mutations) coexisting with that characteristic of histiocytosis (such as *BRAF*^*V600E*^ and *MAP2K1* mutation) were frequently detected by molecular analysis [3, 15]. The *CALR* mutation and the *BRAF*^*V600E*^ mutation were detected in our patient. This finding furthers our understanding of ECD etiology and disease classification and plays a vital role in guiding treatment [3]. Papo et al. reported that there are different kinase mutations in histiocytosis and myeloid tumors, and the use of BRAFIs may promote the growth of malignant tumors driven by mutations other than *BRAF*^*V600E*^ [3], but if they share the same kinase mutation, targeted therapeutics will result in beneficial responses across both conditions. Therefore, evaluation of a concomitant myeloid neoplasm will be critical before initiating targeted therapies for refractory ECD. The guidelines also point out that clinicians should continually monitor peripheral blood counts and strongly consider bone marrow evaluation with myeloid NGS to assess for abnormalities [2, 31]. However, myeloid tumors can be diagnosed before, at the same time as, or after ECD, making these cases a diagnostic challenge for the practicing pathologist.

In conclusion, owing to the rarity and diverse clinical manifestations of ECD, misdiagnosis and missed diagnosis can easily occur. Surgical intervention to obtain tissue samples for definitive diagnosis is crucial, and identification of *BRAF* and other MAPK pathway mutations in biopsies has become extremely helpful in diagnosing the disease. Despite the surgical resection is not curative, its significance lies in biopsy to establish diagnosis and/or surgical debulking to relieve mass effect, minimizing further impairment of visual function. Targeted therapy is the most effective treatment for patients with a positive *BRAF* mutation. In addition, approximately 10.1% of patients with ECD have an overlapping myeloid neoplasm, and myeloid tumors can be diagnosed before, at the same time as, or after ECD. Because the two may share the same pathogenesis, coexisting ECD and myeloid neoplasm may promote or counteract the effect of targeted therapy. Therefore, evaluation of a concomitant myeloid neoplasm is also critical before initiating targeted therapies for refractory ECD.

## Data Availability

The datasets used and analyzed during the current study are available from the corresponding author upon reasonable request.

## Abbreviations

ECD: Erdheim–Chester disease
BRAF: V-raf murine sarcoma viral oncogene homolog B1
CALR: Calreticulin
PLT: Platelet
CT: Computed tomography
MRI: Magnetic resonance imaging
MAPK: Mitogen-activated protein kinase
BRAFIs: BRAF inhibitors
HM: Hand movement
NLP: No light perception
GPA: Granulomatosis with polyangiitis
IgG4-RD: IgG4-related disease
FDA: United States Food and Drug Administration
JAK2V617F: Janus Kinase 2 V617F
MAP2K1: Mitogen-activated protein kinase kinase 1
RDD: Rosai-Dorfman disease
RPE: Retinal pigment epithelium
EDI-OCT: Enhanced depth imaging-optical coherence tomography
